# Integrating WHO thinking healthy programme for maternal mental health into routine antenatal care in China: a randomized-controlled pilot trial

**DOI:** 10.3389/fgwh.2024.1475430

**Published:** 2025-01-06

**Authors:** Anum Nisar, Juan Yin, Jingjun Zhang, Wenli Qi, Jie Yu, Jiaying Li, Xiaomei Li, Atif Rahman

**Affiliations:** 1 Department of Primary Care and Mental Health, Institute of Population Health, University of Liverpool, Liverpool, United Kingdom; 2 School of Nursing, Dalian University, Dalian, China; 3 School of Nursing, Xi’an Jiaotong University, Xi'an, China; 4 Department of Obstetrics, Xi'an Aerospace General Hospital, Xi'an, China; 5 Department of Obstetrics, Xi'an Workers’ Union Hospital, Xi'an, China

**Keywords:** perinatal depression, thinking healthy programme (THP), China, perinatacal care, mental health

## Abstract

**Background:**

Women with perinatal depression and their children are at increased risk of poor health outcomes. Integrating evidence based non-stigmatizing interventions within existing health systems is crucial to reducing psychosocial distress during pregnancy and preventing perinatal depression. This study aimed to evaluate the feasibility of the World Health Organization (WHO) endorsed cognitive-behavior therapy-based Thinking Healthy Programme (THP), delivered by antenatal nurses in China.

**Methods:**

A two-arm pilot randomized controlled pilot trial was conducted to assess the feasibility and of the adapted Chinese version of the Thinking Healthy Programme (THP) among various stakeholders. We recruited pregnant women between 25- and 34-weeks' gestation from two pregnancy schools within the two public sector Hospitals in Xian. Participants in the intervention group attended five face to face sessions of THP facilitated by antenatal nurses. This intervention used cognitive behavior therapy principles to offer psychoeducation, behavioral activation, problem-solving strategies, and social support. In the control group, participants received standard care, which included routine pregnancy education classes led by antenatal nurses. We assessed depressive symptoms using the Patient Health Questionnaire-9 (PHQ-9) at baseline, after the intervention and 4–6 weeks post-intervention, along with evaluations of anxiety, perceived social support, and health-related quality of life.

**Results:**

Among the 737 pregnant women screened, 267 (30.26%) scored ≥5 on the PHQ-9. Out of these, 85 were eligible and consented to participate, with 42 assigned to the intervention group and 43 to the control group. Eighty participants (94.1%) completed the final assessments. Our primary findings indicated that this nurse-delivered intervention was feasible to integrate into routine antenatal care and was well-received by both the women and the delivery agents. Although the study was not designed to detect differences between the intervention and control groups, we observed positive trends towards reductions in anxiety and depressive symptoms favoring the intervention arm. No serious adverse events were reported. This trial is registered in the Chinese Clinical Trial Registry with the registration number ChiCTR1900028114.

**Conclusions:**

We conclude that this intervention, grounded in the well-established WHO Thinking Healthy Programme, is both feasible and acceptable to stakeholders. It merits a definitive randomized trial to assess its effectiveness and cost-effectiveness across various settings.

**Clinical Trial Registration:**

## 1 Introduction

Perinatal depression is generally defined as a depressive episode that occurs during pregnancy or up to 12 months after delivery (1). It is considered an important but unaddressed global health priority (2). In low-and-middle income countries, the prevalence of perinatal depression is estimated to be 12%–25% (3, 4). Untreated perinatal depression may lead to severe adverse health outcomes for both mothers and their children (5). Evidence shows that mothers with perinatal depression have an increased risk of malnutrition, substance abuse, suicide, premature delivery and other adverse pregnancy outcomes (6). Additionally, untreated perinatal depression may lead to developmental problems in the fetus, low birth weight, and increased risk of neonatal intensive care admission (7); increased mortality risk (8); future behavioral disorders and mental health issues in children (9). Perinatal depression can be treated effectively with psychological interventions such as cognitive behavioral therapy. However, over 90% of women in low- and middle-income countries remain untreated due to scarcity of trained human resources to provide such interventions (10). Even in developed countries, it is one of the most frequently underdiagnosed pregnancy complications (11). With high prevalence and low diagnoses rate, perinatal depression has become a significant cause of disease burden globally. It is reported that an estimated 20 million dollars were spent annually on complications arising from untreated prenatal depression (12). Another study from the UK showed that the total lifetime costs of perinatal depression amount to £6.6 billion (13).

Psychosocial interventions including cognitive behavioral therapy and interpersonal therapy are deemed to be effective in preventing perinatal depression (14) and are recommended by many WHO and other health organizations. However, the scarcity of mental health specialists hinders the large-scale use of such interventions. Shifting tasks to trained non-specialists has becoming an expanding area of public mental health research. The Thinking Healthy Programme (THP), first developed in Pakistan, is a cognitive behavior therapy-based intervention for perinatal depression which can be delivered by trained non-specialist providers (15, 16). Our previous research has proved the effectiveness of this psychological intervention in the management of perinatal depression, and the additional investment for scaling up this intervention for perinatal depression in MNCH services was estimated to cost only USD 0.1–0.2 per person per year (15). THP has been adopted by the World Health Organization (WHO) as a part of the WHO's flagship Mental Health Gap Action Programme (mhGAP) for global dissemination (10). In the last decade, THP has been adopted successfully in several countries (17).

China has seen remarkable economic development in economics and urbanization, leading to fast-paced life and more stress for many people. Based on our systematic review (18), the prevalence of perinatal depression showed a significant increasing trend in the last decade, with pooled prenatal depression prevalence of 19.7% and postnatal of 14.8%. Based on National Bureau of Statistics of China 2020 (19), there are 14.65 million newborns in 2019, which translates into more than 4 million mothers with perinatal depression. With the adjustment of birth control policy in recent years (a couple may have three children), higher birth rate is expected (20). However, there is a huge treatment gap in China due to limited mental health resources, with only 1.7 psychiatrists and 0.05 mental hospitals per 100 thousand people (21). In China, it is a medical routine that pregnant mothers go to hospitals for regular medical checks (every two weeks during the second trimester and every week during the third trimester), and the rate of hospital delivery was reported as 99% (22). Moreover, most hospitals in China have established pregnancy schools to provide health education. Therefore, training obstetric nurses in hospitals to deliver the THP may be a potential way of managing perinatal depression (23, 24). Given that antenatal care in China is predominantly hospital-based, utilizing hospital-based nurses allows for smoother integration of the intervention into routine antenatal visits, leveraging nurses' regular interactions with pregnant women in these settings (24). In our previous study, we translated and adapted THP to ensure it is culturally relevant, acceptable, and well-received by the targeted audience, facilitating its integration into the maternal healthcare services of China (25). This study aimed to evaluate the feasibility of integrating nurse-delivered WHO Thinking Health Programme (THP) into hospitals for managing perinatal depression in China.

## 2 Methods

### 2.1 Study design

This study is a two-arm individual-randomized trial, with participants allocated to each arm in a 1:1 ratio to either the intervention arm, which received the Thinking Healthy Programme (THP) sessions, or the control arm, which received standard antenatal care.

### 2.2 Study setting and participants

The study was conducted in Xi'an, the capital of Shaanxi province, an important cultural, industrial, and educational center in central-northwest China. Xi'an has a population of 8 million, with approximately 55,000 nurses primarily employed in large public hospitals. The study took place in typical public sector hospitals in Xi'an, integrating routine care with the pilot intervention program. Two representative public sector hospitals, Xi'an Aerospace General Hospital and Xi'an Workers' Union Hospital, were selected for this research.

The study included women attending the maternity departments at these hospitals who met the following inclusion criteria: they were pregnant between 25- and 34-weeks' gestation, had a score of ≥5 on the Patient Health Questionnaire (PHQ-9), and were willing to participate in the program after providing informed written consent. Women were excluded if they were diagnosed with severe mental disorders such as schizophrenia, mood disorders, or organic disorders; had an intellectual disability or another condition that might affect learning ability; were undergoing psychotherapy or any other psychological intervention; had serious medical conditions requiring inpatient or outpatient treatment, including pregnancy-related complications; scored ≥ 20 on the PHQ-9; or were positive for suicidal ideation (item 9 of PHQ-9).

The study adhered to the ethical standards outlined in the Declaration of Helsinki for research involving human subjects. The study protocol was approved by the relevant ethics review boards, and all participants provided informed consent prior to their involvement. Women attending the maternity departments were informed about the study, and those interested were screened for eligibility. Eligible participants were invited to join the study and provided informed consent. All personal data were kept strictly confidential, with phone numbers used only for session reminders and assessments by research assistants.

### 2.3 Sample size

As this was a pilot feasibility study, detailed power calculations were not conducted.

Our sample size calculations were based on the mean difference of PHQ-9 scores between the two arms post-intervention. Based on a previous study in Pakistan (15), we expected at least 0.724 mean difference between the two groups. A sample size of 72 mothers (36 per arm) provided 85% power to detect this effect size with a 0.05 two-sided alpha level. To account for possible attrition, our final sample size was 84.

### 2.4 Randomization and masking

Mothers who met the criteria and signed the consent form were assigned to either intervention group or control group by random numbers generated from computerized randomizing number generator. The allocation rate was 1:1. The allocations were kept in sealed opaque envelops which were consecutively numbered. The entire randomization scheme was provided by an independent research assistant who was not involved in the study. One designated obstetric nurse enrolled participant and those eligible were assigned to each group based on the number in the envelopes. Masking of participants and nurses who delivered the interventions was not feasible due to the nature of psychosocial intervention. The primary and secondary outcomes of this trial were scores of self-rating scales and were collected by a questionnaire sent to them through WeChat. The researcher who analyzed the data was not aware of the assignments.

### 2.5 Intervention

The intervention is based on the Thinking Healthy Programme (THP) (15, 26, 27), an evidence-based intervention that employs cognitive behaviour therapy (CBT) strategies to achieve three main goals: (a) identifying and modifying maladaptive thinking and behaviours, particularly those leading to poor self-esteem, inability to care for infants, and disengagement from social networks; (b) behavioural activation, which includes adopting behaviours such as self-care, attention to diet, and positive interactions with the infant, and practicing these between sessions; (c) problem-solving to overcome barriers to practicing these strategies. The adapted THP utilizes similar strategies aimed at the prevention rather than the treatment of perinatal depression (25). An additional element is the support provided by the facilitator. The adapted THP consisted of 5 sessions, with each session lasting 45–60 min.

The intervention consists of five sessions: (1) engagement and introduction to the program; (2) psychoeducation and problem-management skills; (3) focusing on personal health and well-being; (4) establishing the mother-infant bond; and (5) reactivating relationships with others and concluding the therapy. The first session was delivered within two weeks after recruitment, sessions 2–4 were delivered weekly or fortnightly. Session 5 was delivered about 40 days after delivery. On average, the intervention sessions lasted one hour, except the introductory session which lasted 90 min. The adapted Mandarin version of THP was designed to be integrated into routine antenatal education classes delivered by nurses who do not have formal mental health care training. The program is fully manualized and includes instructions for delivering each session with culturally appropriate pictorial illustrations to reinforce key messages and encourage family involvement.

The Bernal Framework for adaptation was used to adapt THP for universal use in Chinese hospital settings (28). The original THP manual was translated into Chinese and adapted for the five sessions of antenatal pregnancy school (25). Original pictures were replaced with culturally appropriate illustrations. In total, 15 h of training (spread over 4 days) were conducted by the THP trainers. The training aimed to: (a) educate the nurses on psychosocial factors impacting mother and child health during the perinatal period; (b) teach and practice basic counselling skills; and (c) understand the intervention principles, contents, and delivery mechanisms. Different training methods were used, including lectures, discussions, activities, case scenarios, sharing personal experiences, and role-plays to practice counselling skills and dealing with challenging situations. The intervention material, consisting of job aids, was provided to the nurses to assist them in delivering the intervention. The intervention group received both the routine antenatal sessions and an additional one-hour THP sessions. Full details of the intervention are available in the adaptation paper (25). For the convenience of mothers, all appointments were arranged in the education room in the maternity clinics of the hospital, after their regular medical checks. Mothers with PHQ-9 score above 20 at any time during the intervention were referred to a psychologist or psychiatrist. The adapted THP was shown to be feasible and culturally acceptable to the Chinese population in previous studies (25).

### 2.6 Treatment as usual

Participants randomized to the control arm attended the five sessions of the routine educational group pregnancy classes, facilitated by a designated nurse with no formal training in psychological interventions. These routine pregnancy classes provided online access to education about pregnancy, birth, and new born care. They also included basic information about perinatal depression (PND), how to identify it, and how to seek help. The women in the control arm were able to access all usual care and support offered by the participating hospitals.

### 2.7 Intervention fidelity

The THP was delivered by trained nurses who worked in the maternity department of the two hospitals. The competence of the nurses in delivering THP was assessed post-training using the Enhancing Assessment of Common Therapeutic factors (ENACT), a validated tool that has been developed to evaluate the performance of non-specialist providers on a range of psychotherapeutic domains (29). Intervention fidelity was ensured through weekly supervision of the antenatal nurses by trained THP supervisors. The nurses received group supervision lasting up to 2 h, which involved reviewing the progress of intervention delivery and additional refresher training on intervention components. The THP supervisors, in turn, received monthly supervision from a Master Trainer for approximately 1 h. Additionally, antenatal nurses had day-to-day support from their supervisor at the hospital. Intervention fidelity was tested through independent observations of sessions by the THP supervisors using the Enhancing Assessment of Common Therapeutic Factors tool (ENACT) (29).

### 2.8 Outcome assessments

The primary outcome was the severity of depressive symptoms and was assessed by the Chinese version Patient Health Questionnaire (PHQ-9), with its Cronbach α 0.86 (30, 31). The secondary outcomes included anxiety, perceived social support, and health-related quality of life. Anxiety was assessed by the Chinese version Self-rating Anxiety Scale (SAS), with its Cronbach α 0.931 (32). The SAS was developed by Zung (33). Perceived social support assessed by the Chinese version the Perceived Social Support Scale (PSSS), with its Cronbach α 0.92 (34, 35). Health-related quality of life (HRQOL) was assessed by the EuroQol Five Dimensions Questionnaire (EQ-5D, Chinese version), with its ICC 0.987 (36, 37). The baseline data of all mothers was collected after enrolment by a questionnaire sent to them through the internet and all mothers were assessed again at the completion of the intervention at 4–6 weeks post intervention, using the same questionnaires. Besides, mothers in the intervention group were assessed by PHQ-9 after each session to monitor their depressive symptoms.

### 2.9 Data analysis

SPSS 25.0 software was employed for statistical analysis. Frequencies, percentages, means, and standard deviations were used for statistical description. Independent sample t test and chi-square test were used for statistical inference. Generalized estimating equation (GEE) was employed to evaluate the dynamic changes of each outcome, including the effect of the intervention, time, and their interaction effect. *post hoc* comparisons were conducted to examine whether there were any significant between-group differences based on the estimated marginal means derived from the GEE models. The alpha level was set at 0.05, and the difference was statistically significant with *P* < 0.05.

## 3 Result

In total, 737 pregnant mothers were screened with the PHQ-9 questionnaire and 30.26% of mothers met the inclusion criteria (PHQ-9 score above 5). Among them, 7.46% had PHQ-9 scores above 9. The average score was 4.49 ± 3.21, with the minimum score being 0 and the maximum being 23. Details are shown in Supplementary Appendix SI.

**Figure 1:**
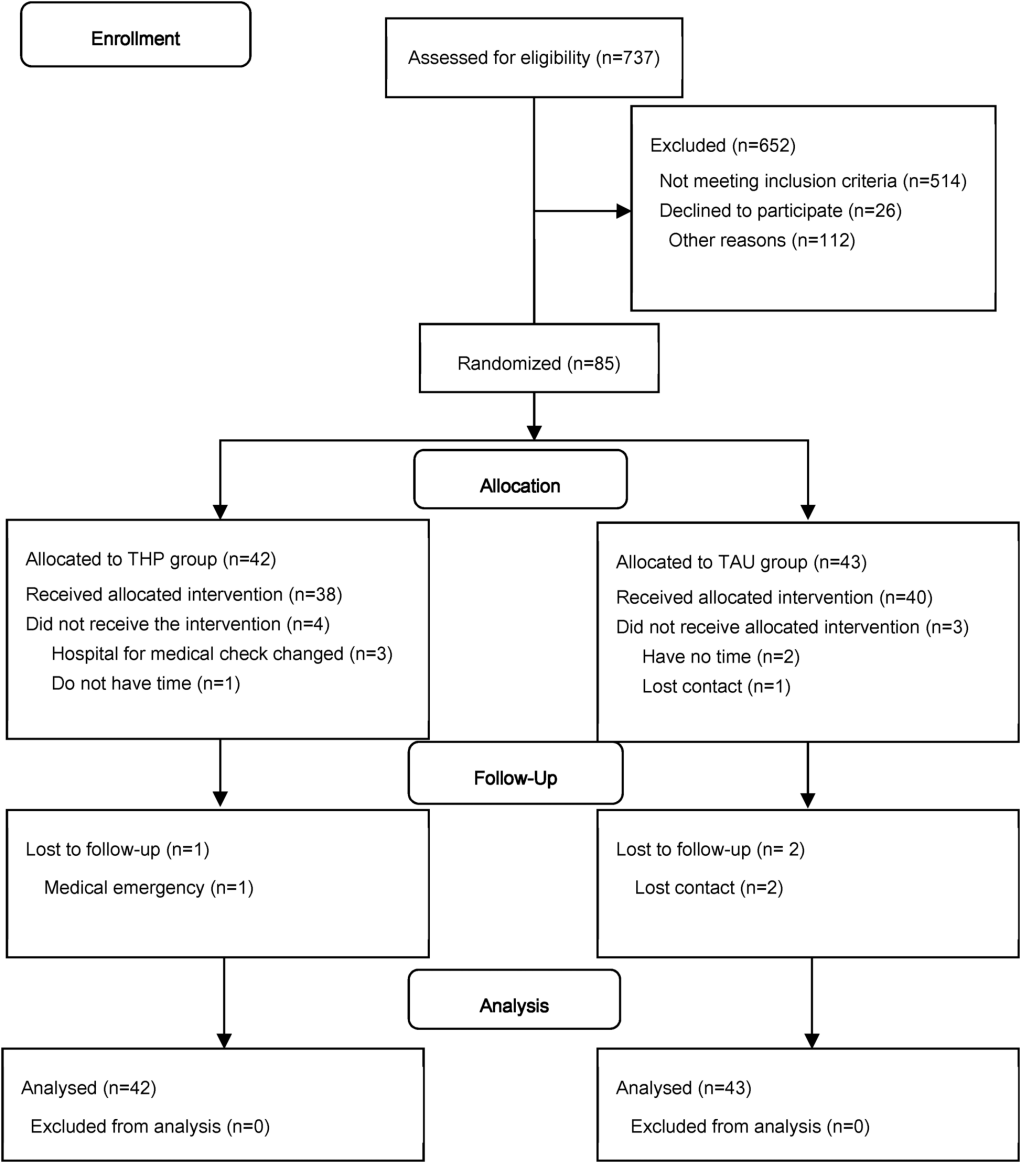
Flow of participants according to CONSORT-2010 format.

Eighty-five mothers were eligible and randomly assigned to the intervention group (*n* = 42) or control group (*n* = 43). In the intervention arm, 38 mothers received the allocated intervention and 37 mothers completed the follow-up. In the control arm, 40 mothers received usual care and 38 mothers completed the follow-up. The flow of participants is shown in Figure 1. The demographic data between the two groups were comparable at baseline (Table 1).

**Table 1:** Characteristics of all participants at baseline.

Characteristics	Intervention group (*n* = 42)	Control group (*n =* 43)
Age, mean (SD)*	28.26 (4.01)	29.02 (4.28)
Gestational age, mean (SD)*	31.17 (3.36)	30.16 (2.92)
Marital status, *n* (%)^#^		
Married	42 (100)	43 (100)
Educational level, *n* (%)^#^		
Senior high school or below	11 (26.19)	19 (44.19)
Junior college and bachelor	30 (71.43)	23 (53.49)
Master's degree or above	1 (2.38)	1 (2.32)
Employment status, *n* (%)^#^		
Full time working	14 (33.33)	8 (18.61)
Part time working	14 (33.33)	24 (55.81)
Unemployed	14 (33.33)	11 (25.58)
Monthly family income, *n* (%)^#^		
≤3,600 RMB per month	13 (30.95)	14 (32.56)
3,600–7,000 RMB per month	20 (47.62)	18 (41.86)
7,000–14,000 RMB per month	7 (16.67)	8 (18.60)
≥14,000 RMB per month	2 (4.76)	3 (6.98)
Parity, *n* (%)^#^		
Multiparous	19 (45.24)	25 (58.14)
Primiparous	23 (54.76)	18 (41.86)
Abnormal pregnancy history, *n* (%)^#^		
Yes	8 (19.05)	10 (23.26)
No	34 (80.95)	33 (76.74)
Health Issues, *n* (%)^#^		
Gestational diabetes	7 (16.6%)	8 (18.6%)
Anemia	4 (9,5%)	5 (11.6%)
Others	6 (14.2%)	7 (16.2%)
None	25 (59.52)	23 (53.49)
Expectations for the baby's gender, *n* (%)^#^		
Yes	14 (33.33)	13 (30.23)
No	28 (66.67)	30 (69.77)
Planned pregnancy, *n* (%)^#^		
Yes	24 (57.14)	28 (65.12)
No	18 (42.86)	15 (34.88)
Desired mode of delivery, *n* (%)^#^		
Vaginal delivery	20 (47.62)	24 (55.82)
Cesarean section	5 (11.90)	4 (9.30)
Not Mind	17 (40.48)	15 (34.88)

* *t*-test.

# Chi-square test.

**Figure 2:**
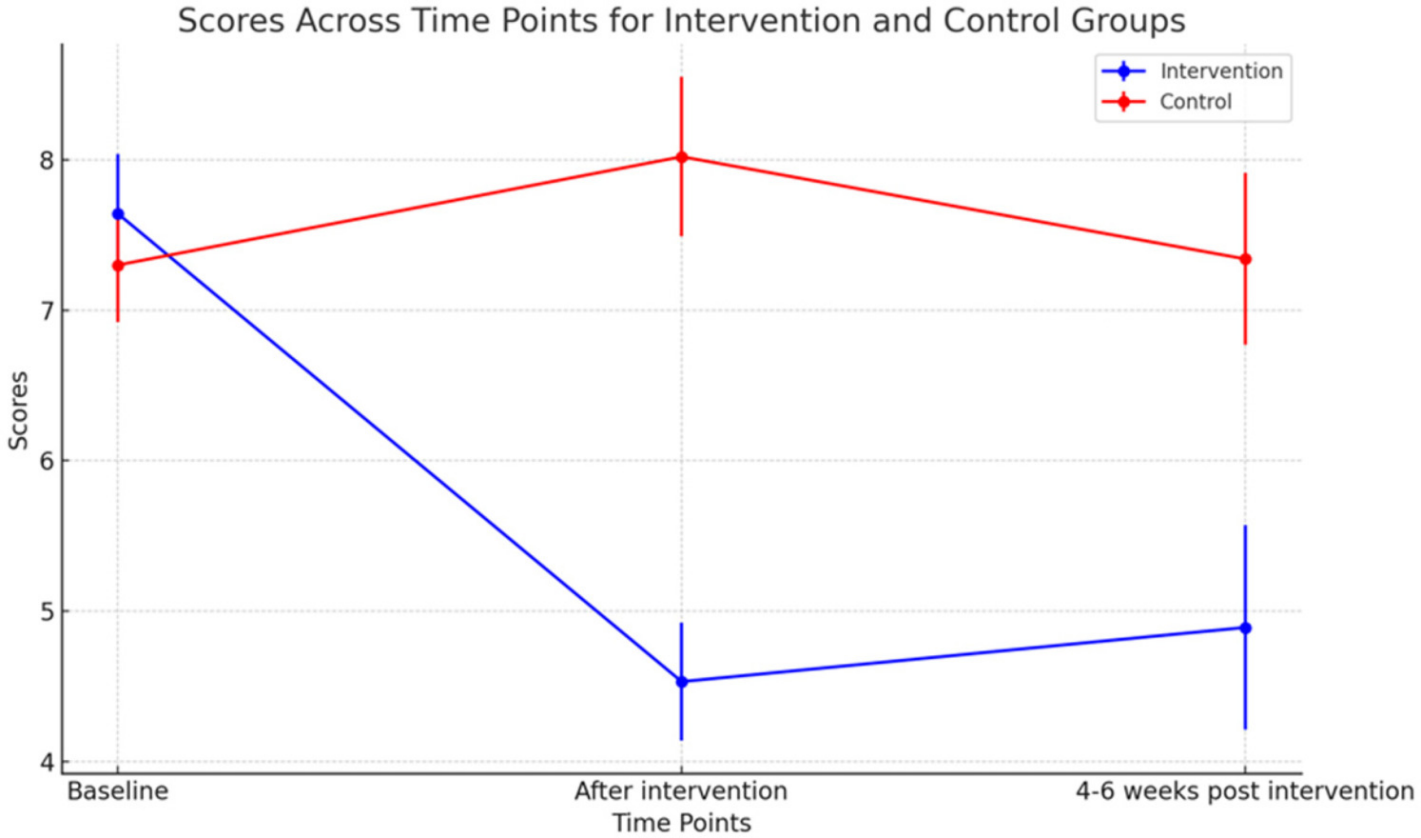
PHQ-9 scores over time in the intervention vs. control arm.

At baseline, the depressive symptoms between the two groups were not significantly different (*P* = 0.533). After the intervention, the depressive symptoms of the THP group were much lower than those of the control group (M diff = −3.50, *P* < 0.001). Although there was a slight increase in PHQ-9 scores in the THP arm at 4–6 weeks post intervention compared to after the intervention, they were still significantly lower than those of the control arm (M diff = −2.45, *P* = 0.006). The details are shown in Table 2 and Figure 2.

**Table 2:** Effects of the intervention on PHQ-9 by groups across time using GEEs.

	Intervention mean (SE)	Control mean (SE)	Between-group comparison	
			M diff (95% CI)	*P*
Baseline	7.64 (0.40)	7.30 (0.38)	0.34 (−0.73 to 1.41)	0.533
After intervention	4.53 (0.39)	8.02 (0.53)	−3.50 (−4.79 to −2.21)	<0.001
4–6 weeks post intervention	4.89 (0.68)	7.34 (0.57)	−2.45 (−4.20 to −0.70)	0.006

Mdiff, difference in the estimated marginal means.

For mothers in the THP group, the PHQ-9 scores dropped continuously after each session. However, the scores increased slightly at the second follow-up compared to the scores after the intervention.

For secondary outcomes, the baseline data indicated that the anxiety symptoms, perceived social support, and HRQOL of the two groups were comparable. Post-intervention, the anxiety scores in the THP group decreased slightly and were significantly lower than those of the control group (Table 3), both immediately after the intervention (M diff = −5.28, *P* < 0.001) and 4–6 weeks post intervention (M diff = −3.99, *P* = 0.013).

**Table 3:** Effects of the intervention on SAS by groups across time using GEEs.

	Intervention mean (SE)	Control mean (SE)	Between-group comparison	
			Mdiff (95% CI)	*P*
Baseline	47.14 (1.05)	47.26 (0.84)	−0.12 (−2.76 to 2.52)	0.928
After intervention	41.77 (0.79)	47.05 (0.86)	−5.28 (−7.58 to −2.98)	<0.001
4–6 weeks post intervention	42.15 (1.13)	46.15 (1.15)	−3.99 (−7.15 to −0.83)	0.013

Mdiff, difference in the estimated marginal means.

Mothers in the THP group perceived more social support than those in the control group (Table 4), as illustrated by a higher mean score on the PSSS (M diff = 3.83, *P* = 0.076). This effect lasted until 4–6 weeks post intervention (M diff = 3.65, *P* = 0.190).

**Table 4:** Effects of the intervention on PSSS by groups across time using GEEs.

	Intervention mean (SE)	Control mean (SE)	Between-group comparison	
			Mdiff (95% CI)	*P*
Baseline	60.29 (1.85)	60.44 (1.57)	−0.16 (−4.91 to 4.59)	0.949
After intervention	63.61 (1.47)	59.78 (1.59)	3.83 (−0.41 to 8.07)	0.076
4–6 weeks post intervention	62.68 (1.54)	59.03 (2.32)	3.65 (−1.80 to 9.10)	0.190

Mdiff, difference in the estimated marginal means.

However, no significant differences were observed between the two groups regarding health-related quality of life at both time points (M diff = 4.76, *P* = 0.036; M diff = 4.09, *P* = 0.247). Details can be found in Table 5.

**Table 5:** effects of the intervention on EQ-5D by groups across time using GEEs.

	Intervention mean (SE)	Control mean (SE)	Between-group comparison	
			Mdiff (95% CI)	*P*
Baseline	78.79 (2.36)	78.23 (2.45)	0.55 (−6.11 to 7.22)	0.871
After intervention	85.13 (1.40)	80.38 (1.78)	4.76 (0.32 to 9.20)	0.036
4–6 weeks post intervention	84.59 (1.78)	80.50 (3.06)	4.09 (−2.84 to 11.03)	0.247

Mdiff, difference in the estimated marginal means.

## 4 Discussion

Given the successful achievement of recruitment and randomization, low levels of attrition at follow-up, and effective delivery of THP, our findings indicate that this the intervention is feasible for integration into routine antenatal educational classes and was well-received by the participating women. The findings indicated that the nurse-led THP notably reduced depression and anxiety symptoms among pregnant mothers' post-intervention, with the improvements continuing into the postpartum period.

Our findings contribute to the recent global review of evidence conducted by the World Health Organization (WHO), which highlights moderate to strong effect sizes favoring preventive interventions for reducing the severity of depressive and anxiety symptoms during the postnatal period (27, 38). These reviews found that the most significant effects were seen in interventions targeting high-risk groups during the antenatal period, with cognitive behavior therapy being the most commonly used approach. Based on this evidence, the WHO guidelines recommend implementing preventive interventions during the antenatal period as part of maternal health care to prevent postnatal depression (39). However, the WHO notes that most evidence for preventive interventions comes from high-income countries and calls for further research in low- and middle-income countries (LMICs).

Our study also showed that THP led to significant improvements in the perceived social support of mothers from friends, family members and partners. A previous qualitative study conducted in China identified showed that the disparity between anticipated and available social support, and conflicts among support providers were two significant stressors associated with postpartum depression (40). With rapid urbanization and consequent changes to family structure and traditional support systems, mothers may encounter difficulties in coping with the demands of motherhood which might contribute to anxiety and depressive symptoms. One key aspect of THP is helping mothers with their relationship with significant others and thus could increase their social support from significant others. In a previous THP study conducted in Pakistan, mothers also showed better perceived social support at 6 and 12 months follow-up, compared with the control group (15).

Mothers reported improved quality of life in the THP group immediately after intervention, but this was insignificant on follow-up at 40 days postpartum. A recent systematic review suggests that psychotherapies might improve maternal quality of life during the antenatal stage of pregnancy (41) but longer sustained changes remain uncertain (42–44). Evidence showed that depression, anxiety, and social support are important contributors to quality of life (45), and the alleviation of these indicators may improve this in pregnant women. However, HRQOL is a very comprehensive index and many other factors are likely to contribute (46, 47). This indicates that in addition to psychosocial interventions, other important social, interpersonal, and economic factors may also need to be addressed to bring about sustained improvements in quality of life.

Our study has several limitations. It has a small sample size and was conducted in one geographical area of Xi'an which is largely urban. We cannot generalize the results to other populations and larger studies from more diverse populations may be needed. We did not monitor the long-term effect of our intervention and although short-term outcomes of the study were optimistic, the long-term effect remained unclear. Two other studies from India and Pakistan showed that differences between intervention and control groups reduced at 6 months follow-up (48, 49). Further studies are needed to explore the long-term effect of THP in China. We did not evaluate the cost-effectiveness of the intervention or evaluate the burden of delivering the intervention on the nursing staff. This needs to be addresses in future studies. While initial work has established the intervention's acceptability, future studies should include qualitative interviews to gain deeper insights into participant experiences and pinpoint areas for enhancement.

In conclusion, Thinking Healthy Programme is feasible and acceptable to stakeholders and warrants a definitive randomized trial to evaluate its effectiveness and cost-effectiveness in China. However, future large-scale and innovativly designed studies are needed to further test this effect, measure long-term outcomes, and evaluate cost-effectiveness, optimal dosage and timing and implementation challenges of delivering the intervention through nurses.

## Data Availability

The raw data supporting the conclusions of this article will be made available by the authors, without undue reservation.
