# Supplementary material for: Integrating WHO thinking healthy programme for maternal mental health into routine antenatal care in China: a randomized-controlled pilot trial
Source: Front Glob Womens Health. 2025 Jan 6;5:1475430. doi: 10.3389/fgwh.2024.1475430 (PMC11743642; doi:10.3389/fgwh.2024.1475430)
Supplement: Supplementary file 1 [file Table1.docx]

**Appendix I**

**Details of PHQ-9 scores in screening (n=737)**

| Item | Score=0  (%) | Score=1  (%) | Score=2  (%) | Score=3  (%) | Mean  (SD) |
| --- | --- | --- | --- | --- | --- |
| Little interest or pleasure in doing things? | 43.7 | 49.7 | 4.7 | 1.9 | 0.65(0.66) |
| Feeling down, depressed, or hopeless? | 51.6 | 44.5 | 2.4 | 1.5 | 0.54(0.62) |
| Trouble falling or staying asleep, or sleeping too much? | 29 | 57.5 | 9.4 | 4.1 | 0.88(0.73) |
| Feeling tired or having little energy? | 29.7 | 57.7 | 9.1 | 3.5 | 0.86(0.72) |
| Poor appetite or overeating? | 54.1 | 37.6 | 6 | 2.3 | 0.53(0.71) |
| Feeling bad about yourself - or that you are a failure or have let yourself or your family down? | 80.9 | 16.8 | 1.6 | 0.7 | 0.22(0.50) |
| Trouble concentrating on things, such as reading the newspaper or watching television? | 69.6 | 24.2 | 4.9 | 1.4 | 0.38(0.64) |
| Moving or speaking so slowly that other people could have noticed? Or the opposite - being so fidgety or restless that you have been moving around a lot more than usual? | 73 | 21.3 | 3.8 | 1.9 | 0.35(0.65) |
| Thoughts that you would be better off dead, or of hurting yourself in some way? | 96.3 | 3.3 | 0 | 0.4 | 0.04(0.26) |
